# Ascites—VII

**Published:** 1909-07-24

**Authors:** 


### ASCITES—VII.

### THE DIFFERENTIAL DIAGNOSIS OF THE CAUSE OF ASCITES (*Continued*).

Ascites, when due to obstruction of the portal vein by pressure or invasion from outside, is generally associated with intense jaundice, owing to the simultaneous obstruction to the common bile duct or the hepatic ducts. Tumours may be felt, but great difficulty is often experienced in accurately determining their nature and origin.

Tumours of the liver or of the gall bladder are associated in the majority of cases with hepatic enlargement. Careful examination of the edge and surface of the liver under such circumstances may adduce valuable evidence as to the cause of its increase in size.

Tumours of the head of the pancreas may not only cause deep and persistent jaundice, but they also lead to enormous dilatation of the gall bladder; the enlargement of the latter may be as great in cases of carcinoma of the head of the pancreas as in any other condition whatever. In addition to this there may be glycosuria, and the stools may contain excess of fat and many undigested muscle fibres. Even if glycosuria itself does not occur, the urine may give a strongly positive Cammidge's reaction. On account of the close relation of the pancreas to the aorta, a well-marked transmitted pulsation may be felt in a pancreatic tumour. If the stomach is inflated it may be possible to demonstrate that the tumour lies posterior to this viscus, a sign of much diagnostic importance.

Tumours of the kidney may be associated with hæmaturia, albuminuria, pyuria, and the presence of abnormal cells, or even obvious fragments of new growth in the centrifugalised urinary deposit. When large, a renal tumour of the right side is apt to become attached to the under surface of the liver, in which case it becomes difficult of distinction from a hepatic enlargement or from a big dilated gall-bladder. On the left side a splenic enlargement may be simulated. We have dealt with the differential diagnosis between renal and splenic tumours in a former article.

Carcinoma of the stomach, if it were the direct cause of ascites—as distinct from causing it indirectly after leading to secondary deposits in the peritoneum, or in the portal glands—would most likely be situated at the pyloric end, and would thus give rise to pyloric obstruction and gastrectasis, with copious vomiting at intervals, and with all the familiar physical signs of dilated stomach. Enlarged glands would be secondary to changes in other organs. Pathological curiosities, such as aneurysm of the hepatic artery obstructing the portal vein and thus causing ascites, need not be discussed.

*Thrombosis of the Portal Vein.*—In addition to obstruction from without the portal vein may become partially or wholly occluded by thrombosis from pylephlebitis. The diagnosis of this condition is decidedly difficult. It has to be distinguished from the other forms of portal obstruction just mentioned, and also particularly from cirrhosis of the liver. The absence of all history of alcoholism and of the signs it often leads to, and the absence of any other known cause of portal obstruction in a patient who has rapidly developed ascites, considerable enlargement of the spleen, dilatation of the superficial abdominal veins, and possibly hæmatemesis, should suggest the possibility of adhesive pylephlebitis as a diagnosis. In this disease the fluid may reaccumulate as rapidly as under any other circumstances after the abdomen has been tapped.

*Obstruction of the Hepatic Veins or of the Inferior Vena Cava at the level of, or above, these veins* may occur as a result of thrombosis or obliterative inflammation of the hepatic veins or of thrombosis of or pressure on or invasion of the inferior vena cava by tumours at the level of the diaphragm. It must necessarily be a rather rare condition, but it is important because it is so apt to be overlooked. Ascites is invariably produced, and, as in cardiac disease, it is usually preceded by swelling and œdema of the feet and legs. The subcutaneous abdominal veins may be dilated and tortuous with reversal of the normal direction of blood-current in them. The spleen is usually enlarged, and there may be gastro-intestinal hæmorrhages. Œdema may reach up the back to the level of the ribs, and there may be both albumen and blood in the urine, with abundance of renal epithelial cells, owing to stagnation in the renal veins.

(*To be continued.*)

